# Treatment of Permanent Stricture of the Urethra by Sudden Dilitation

**Published:** 1864-03

**Authors:** Wm. B. Slayter

**Affiliations:** Chicago, Graduate Royal College of Physicians of England; Royal Colleges of Surgeons of London and Dublin; Late House-Surgeon Westminister Hospital, London


### TREATMENT OF PERMANENT STRICTURE OF THE URETHRA BY SUDDEN DILITATION.

By WM. B. SLAYTER, M.D., of Chicago,

*Graduate Royal College of Physicians of England; Royal Colleges of Surgeons of London and Dublin; Late House-Surgeon Westminister Hospital, London.*

Of all diseases of the urinary organs which fall to the lot of the Surgeon, permanent stricture of the urethra, perhaps, is the most annoying and tedious to the patient and the most troublesome to the medical attendant, and it is for this reason that I feel called upon to bring before the profession of this city a mode of treatment which has been sanctioned and adopted by many of the principal Surgeons of Great Britain, among whom may be mentioned Professor Fergusson of King's College Hospital, London, Mr. Skey of St. Bartholomews, Mr. Cutter, and numerous others of world-wide reputation.

This treatment consists in a forcible and sudden dilitation of the stricture by means of an instrument invented by Mr. Holt of the Westminister Hospital, and with which, in the space of a few seconds, the largest size catheter may be passed into the bladder without the slightest difficulty.

Strange to say, this treatment has received very little attention in America, and, with the exception of one or two Surgeons in New York--so far as I know—has not been alluded to by any American writer. The instrument itself consists of two portions: first, two solid silver branches of the shape of an ordinary bougie, grooved internally, convex externally, and firmly attached to each other at the point; at the handle is a screw by which they are compressed together to the size of a No. 3 catheter, and between the two is a silver wire which serves as a guide for the straight hollow dilator with which the stricture is burst.

Owing to the size of the instrument it is impossible to use it until the Surgeon has succeeded in passing a No. 3 catheter through the stricture, and then it may be employed in the great majority of cases without the least fear or hesitation. In some cases this treatment has been adopted when only a No. 1 catheter could be passed, but the instrument used has been of smaller dimensions than the ordinary one.

The following, then, is the mode of procedure, after the Surgeon has succeeded in passing a No. 3 catheter: The branches being tightly screwed together, are passed through the stricture into the bladder; they are then unscrewed, and the largest size dilator, No. 10 or 11, is forcibly pushed down through the stricture on the silver guide. The instrument is immediately withdrawn, a No. 10 or 11 catheter passed and the urine drawn off. The patient is directed to take Quinine gr. ij Tinct. Opii. m. x.—three times a day for the first twenty-four hours, for the purpose of preventing any stricture fever coming on, he is also allowed to empty his bladder without the aid of a catheter for the first two days. A No. 11 catheter is again passed on the second and every alternate day for a week or fortnight, when the patient, first being taught, is directed to pass an instrument for himself occasionally.

The *modus operandi* of the instrument has, I think, been conclusively proved by the following experiment, which Mr. Holt quotes in his work on stricture: A man died in Hospital, I believe, from disease of the chest, and at the time of his death was the subject of a very tight stricture. At the post-mortem examination Mr. Holt introduced the dilator into the bladder, split the stricture, and removed the parts. The fibrine forming the stricture was found to be completely burst open, but the mucous membrane lining the urethra remained quite uninjured. The hœmorrhage after this operation is very trifling; in the majority of cases amounting to half-a-dozen drops, and in very rare instances to a teaspoonful.

The great advantages of this over the ordinary mode of treatment by gradual dilitation are: 1st—The rapidity with which a stricture is cured. 2nd—It is less liable to return after this treatment. 3rd—Its perfect freedom from danger.

1st—The rapidity with which a stricture is cured. As I have before stated, any ordinary permanent stricture, provided a No. 3 catheter can be passed, may be cured in a fortnight; whereas, by the ordinary method, it very often takes months before a patient can dispense with the doctor. This saving of time is certainly an immense advantage, especially in cases where fistulæ, abscess, irritation or chronic inflammation of the bladder are threatened, or where these complications are already present. For by removing the stricture, these diseases rapidly get well.

2nd—Stricture is less liable to return after this treatment. Stricture, however treated, is liable to return, but it has been proved most satisfactorily that it is less likely to do so after the fibrine has been burst open than when gradually dilated. In the very few cases in which the stricture has returned, it has been clearly traced either to the patient's neglect in not passing the catheter occasionally, or to his taking cold, or a too free indulgence in drink or other excesses. I have seen very many cases, two and three years after they have been operated upon, and they have never had the slightest return of their complaint or experienced any difficulty from it whatever.

3rd—Its perfect freedom from danger. When this treatment was first brought to the notice of the profession, all sorts of imaginary dangers and difficulties arising from it were conjectured. In the first place it was said that the pain of the operation would be too great for most people to put up with. Mr. Holt at first administered chloroform; but after the first few cases the pain of the operation was found to be so very slight that it was discontinued, and now, except in very rare instances, it is never given. The next objection was that it would necessitate the retirement of the patient from his ordinary business for some time; but experience has shown that three or four hours quiet after the operation is all that is necessary.

Some imagined that this apparently rough treatment might produce abscess of the urethra, extravasation of urine, and even death. Mr. Holt has now operated on upwards of two hundred cases, many of which I have watched, when House-Surgeon at the Westminister Hospital. I also operated on upwards of fifty cases, and the only bad symptoms that have ever been noticed have been a few rigors, and these in a very few cases; and in not a *single instance* has abscess or other serious complication arisen.

In conclusion, I would say, after having had a large experience in all kinds of treatment of stricture, that I believe this, in the majority of cases, to be the very best; and I am satisfied that any Surgeon who gives it a fair trial will be very soon convinced of its superiority and will never have cause to regret having used it.

